# Overlap Between Apolipoprotein Eε4 Allele and Slowing Gait Results in Cognitive Impairment

**DOI:** 10.3389/fnagi.2019.00247

**Published:** 2019-09-13

**Authors:** Ryota Sakurai, Yutaka Watanabe, Yosuke Osuka, Yu Taniguchi, Hisashi Kawai, Hunkyung Kim, Akihiko Kitamura, Hiroki Inagaki, Manuel Montero-Odasso, Shuichi Awata, Shoji Shinkai

**Affiliations:** ^1^Research Team for Social Participation and Community Health, Tokyo Metropolitan Institute of Gerontology, Tokyo, Japan; ^2^Gait and Brain Lab, Parkwood Institute, Lawson Health Research Institute, London, ON, Canada; ^3^Research Team for Promoting Independence and Mental Health, Tokyo Metropolitan Institute of Gerontology, Tokyo, Japan; ^4^Gerodontology, Department of Oral Health Science, Faculty of Dental Medicine, Hokkaido University, Sapporo, Japan; ^5^Research Team for Human Care, Tokyo Metropolitan Institute of Gerontology, Tokyo, Japan; ^6^Department of Medicine, Division of Geriatric Medicine, Schulich School of Medicine and Dentistry, University of Western Ontario, London, ON, Canada

**Keywords:** apolipoprotein E, slow gait, cognitive impairment, aging, older adults, cohort study

## Abstract

**Background**: Although apolipoprotein E polymorphism ε4 allele (ApoE4) and slow gait are well-known risk factors for cognitive impairment, examination of their combined effect on cognitive function is lacking. Our objective was to elucidate whether a combination of ApoE4 phenotyping and slow gait resulted in greater cognitive impairment.

**Methods**: Overall, 1,085 community-dwelling older adults, either ApoE4 carriers (*n* = 167, 15.4%) or non-ApoE4 carriers, were included from the “Takashimadaira study.” Gait speed was assessed with an electronic walkway and slow gait was defined as <1 m/s. Cognitive performance was also assessed using the Mini-Mental State Exam (MMSE) and the Trail Making Test (TMT)-A and -B. A two-way analysis of covariance (ANCOVA; ApoE and gait velocity factors) adjusted for covariates was performed for each analysis.

**Results**: Gait and cognitive performances were similar for ApoE4 and non-ApoE4 carriers. A two-way ANCOVA of the MMSE showed a significant interaction between the two factors. ApoE4 carriers with slow gait had lower MMSE scores than ApoE4 carriers without slow gait and non-ApoE4 carriers with slow gait. Also, a significant main effect of gait velocity on TMT-A was observed, indicating that slow gait is associated with lower scores irrespective of the presence of ApoE4. There was no main effect or interaction observed on the TMT-B.

**Conclusions**: Our results suggest that the concurrent presence of at least one copy of ApoE4 and slow gait can define a subgroup with the lowest cognition. Elucidating the mechanisms underlying these associations may point out modifiable factors in populations at risk of dementia.

## Introduction

Apolipoprotein E polymorphism ε4 allele (ApoE4) is associated with cognitive impairment (Farrer et al., 1997; Liu et al., 2013) and is considered one of the most robust risk factors for Alzheimer’s disease (AD). A previous meta-analysis has shown that the odds of developing AD increases 14.9-fold among ApoE4 carriers compared with individuals without ApoE4 (e.g., E3/E3; Farrer et al., 1997). Similar to ApoE4, slow gait has also been associated with increased risk of cognitive impairment (Verghese et al., 2008; Dumurgier et al., 2017; Montero-Odasso et al., 2019), and it is regarded as a strong predictor of dementia in population-based and clinical studies. As slowing gait often appears earlier than cognitive changes (Camicioli et al., 1998; Montero-Odasso et al., 2016), it may represent a surrogate motor marker for cognitive decline associated with aging and neurodegeneration. However, the combined effect of carrying ApoE4 phenotyping with slow gait speed on cognitive performance is poorly known.

A recent longitudinal study suggests that a combination of ApoE4 and gait slowing is associated with lower memory (Jayakody et al., 2019). Indeed, although ApoE4 is a known genetic risk factor for pathological aging, ApoE4 carriers do not always develop cognitive impairment, including pathological aging (Nadkarni et al., 2017; Sundermann et al., 2018). Accordingly, it is hypothesized that the effect of ApoE4 on cognitive impairment becomes more hazardous when combined with slow gait.

To confirm our hypothesis, we examined the ApoE genotype and gait velocity in a well-characterized large sample of community-dwelling older adults free of dementia. Our goal is to take the first step toward developing an understanding of the relationship between ApoE4 and slow gait on cognitive function in older adults. The expected findings of this study may help detect mild cognitive impairment (MCI) or dementia in early stages.

## Materials and Methods

### Participants

All data were collected from an ongoing prospective cohort study, named the “Takashimadaira Study,” which aimed to assess and promote dementia-friendly communities in a metropolitan area. The design and logistics of this study have been described in detail elsewhere (Taniguchi et al., 2019).

Based on the local resident registration (*n* = 7,614), we conducted an initial survey *via* mail among community-dwelling adults aged 70 years or older between August and September 2016 in a northern city of Tokyo, Itabashi, Japan. After collecting responses from 5,430 participants (71.3%), we mailed them non-mandatory recruitment letters for a health check-up during which they could decide to participate. A total of 1,360 older adults participated in the health check-up between October and December 2016.

The participants were included in the study based on the following criteria: (1) being able to walk independently for 5 min; and (2) completing ApoE genotyping. The exclusion criteria included having: (1) Parkinsonism or any other neurological disorder (e.g., severe stroke) with a residual motor deficit; (2) active osteoarthritis affecting the lower limbs performance; and (3) having dementia assessed by self-reported medical history.

Ethics approval was obtained from the Tokyo Metropolitan Institute of Gerontology Ethics Board, and informed signed consent was obtained from the participants at enrollment prior to study assessments.

### Measurements

#### ApoE4 Genotyping

To determine the ApoE genotype, isoelectric focusing and western blotting were conducted (SRL Inc., Tokyo, Japan). Briefly, serum samples were incubated with neuraminidase to remove sialic acids. Lipoproteins were isolated by precipitation with tungstophosphoric acid and magnesium chloride, and lipids were extracted using ethanol and diethyl ether. The remaining apoproteins were dissolved in tris-dithiothreitol/urea buffer, separated by isoelectric focusing, blotted onto a nitrocellulose membrane, and detected using a specific mouse monoclonal antibody against ApoE. Participants were assigned as either ApoE4 carriers (E4/E2, E4/E3, and E4/E4) or non-ApoE4 carriers (E2/E2, E3/E2, and E3/E3).

#### Gait Assessments

Gait velocity (m/s) was assessed using an electronic walkway (P-Walk, BTS engineering, Italy, 500 cm long). The start and end points for measurement were marked on the floor one meter from both the walkway start and end points. These one-meter markings were used to avoid recording participants’ acceleration and deceleration phases on the walkway. Each participant performed one practice trial walking on the mat at their usual pace. For the recorded walk, participants were again instructed to walk on the walkway at their usual pace. The gait trials occurred in a well-lit room, and participants walked barefoot without any additional attached monitors. To show gait characteristics among our sample, gait variability in stride time and length were also measured. We defined conventional slow gait as <1 m/s based on a prior definition (Studenski, 2009), and then assigned participants into either slow gait or non-slow gait groups.

#### Cognitive Function

To assess global cognition and other cognitive domains (i.e., visual search, motor speed skills, attention, working memory, and task-shifting), participants underwent the Mini-Mental State Exam (MMSE; scores ranging from 0 to 30, with higher scores indicating higher overall cognitive function; Folstein et al., 1975) and the Trail Making Test (TMT)-A and -B (Sánchez-Cubillo et al., 2009). The TMT-A consists of a series of 25 numbered circles, and participants are asked to connect numbers from 1 to 25 in ascending order. The TMT-B consists of 13 numerical numbers and 12 letters, requiring participants to connect numbers and letters alternatively in ascending order.

#### Covariates

Relevant sociodemographic and clinical variables, including age, sex, education level, body mass index (BMI), total cholesterol, depression symptoms, and number of comorbidities were recorded and assessed as covariates of the relationship among the ApoE genotype, gait, and cognitive performances. Depression symptoms were assessed using the Geriatric Depression Scale (GDS; Yesavage and Sheikh, 1986).

### Statistical Analyses

By using ApoE4 carrier status and slow gait status, participants were assigned into four groups as follows: non-ApoE4 carriers without slow gait, non-ApoE4 carriers with slow gait, ApoE4 carriers without slow gait, and ApoE4 carriers with slow gait.

Descriptive statistics of the differences among the four groups were examined using Chi-square tests for the categorical variables and analysis of variance (ANOVAs) for continuous variables. We also performed sub-group *t*-tests on gait variables for non-ApoE4 carriers and ApoE4 carriers to confirm whether having the ApoE4 allele is simply associated with lower gait performance including slow gait.

To examine the cumulative effect of the two factors on cognitive function, two-way factorial analysis of covariances (ANCOVA), including ApoE (non-ApoE4 and ApoE4 carriers) and gait velocity (non-slow gait and slow gait), were performed on the MMSE, TMT-A, and TMT-B. ANCOVA was adjusted for age, sex, education level, BMI, total cholesterol, GDS score, and the number of comorbidities.

Statistical analyses were performed using IBM SPSS Statistics version 23.0 (SPSS Inc., Chicago, IL, USA), with the level of significance set at *p* < 0.05.

## Results

Among 1,360 individuals, 1,085 older adults (mean age 77.1 ± 4.7 years, 60.2% women) were included in this analysis since they completed ApoE genotyping and gait assessment. Distribution of ApoE genotypes were as follows: E2/E2, 0.4%; E3/E2, 7.3%; E3/E3, 77.0%; E4/E2, 0.9%; E4/E3, 13.7%; and E4/E4, 0.7%.

Table 1 shows participant’s characteristics stratified by ApoE4 status and slow gait status. The proportion of female participants was significantly higher in non-ApoE4 carriers without slow gait; and the proportion of cerebrovascular disease was significantly higher in groups with slow gait compared with the groups without slow gait. Also, older age, higher number of comorbidities, and greater GDS score tended to be higher in groups with slow gait. Groups with slow gait naturally showed significant slower gait velocity; furthermore, they showed greater gait variabilities. Sub-group analyses examining the effects of ApoE4 allele on poor gait performance showed that there was no significant difference in gait performances between non-ApoE4 carriers and ApoE4 carriers (Table 2).

**Table 1:** Characteristics stratified by ApoE4 carrier and slow gait statuses.

Variables, mean (SD)	Non-ApoE4 carriers without slow gait (*n* = 807)	Non-ApoE4 carriers with slow gait (*n* = 111)	ApoE4 carriers without slow gait (*n* = 148)	ApoE4 carriers with slow gait (*n* = 19)	*p*-value
Female, *n* (%)	500 (62.0)	58 (52.3)	88 (59.5)	7 (36.8)	0.040
Age	76.8 (4.5)	79.4 (5.4)	76.3 (4.5)	79.1 (6.5)	*p* < 0.001
Number of years of education	12.7 (2.7)	12.6 (2.9)	12.8 (2.7)	13.3 (3.0)	0.619
Body Mass Index	23.1 (11.9)	23.6 (3.3)	22.0 (2.9)	22.1 (3.1)	0.569
Total number of comorbidities	1.49 (1.46)	1.96 (1.61)	1.54 (1.57)	2.11 (1.70)	0.006
Hypertension, *n* (%)	368 (45.6)	60 (54.1)	58 (39.2)	9 (47.4)	0.129
Cerebrovascular disease, *n* (%)	45 (5.6)	17 (15.3)	8 (5.4)	4 (21.1)	*p* < 0.001
Heart disease, *n* (%)	113 (14.0)	16 (14.4)	15 (10.1)	4 (21.1)	0.462
Diabetes mellitus, *n* (%)	95 (11.8)	20 (18.0)	17 (11.5)	4 (21.1)	0.181
Hyperlipidemia, *n* (%)	165 (20.4)	24 (21.6)	42 (28.4)	6 (31.6)	0.128
Respiratory disease, *n* (%)	97 (12.0)	22 (19.8)	20 (13.5)	2 (10.5)	0.145
Depression, *n* (%)	27 (3.3)	6 (5.4)	8 (5.4)	0 (0.0)	0.384
Total cholesterol, mg/dl	211.3 (36.9)	205.6 (39.5)	214.4 (33.2)	203.4 (37.8)	0.218
GDS	3.7 (3.2)	4.9 (3.6)	3.8 (3.4)	4.8 (4.6)	0.002
Gait velocity, m/s	1.32 (0.18)	0.85 (0.15)	1.31 (0.18)	0.85 (0.15)	*p* < 0.001
Stride time variability, (CoV)%	3.08 (1.25)	3.88 (1.49)	3.14 (1.09)	3.73 (1.40)	*p* < 0.001
Stride length variability, (CoV)%	2.56 (1.15)	3.84 (1.63)	2.64 (1.23)	3.69 (1.23)	*p* < 0.001

**Table 2:** Sub-group analysis for difference in gait performance between non-ApoE4 carriers and ApoE4 carriers.

Variables, mean (SD)	Non-ApoE4 carriers (*n* = 918)	ApoE4 carriersVariables, mean (SD) (*n* = 167)	*p*-value
Gait velocity, m/s	1.26 (0.23)	1.26 (0.23)	0.739
Stride time variability, (CoV)%	3.18 (1.32)	3.22 (1.16)	0.718
Stride length variability, (CoV)%	2.73 (1.31)	2.77 (1.26)	0.719

**Figure 1:**
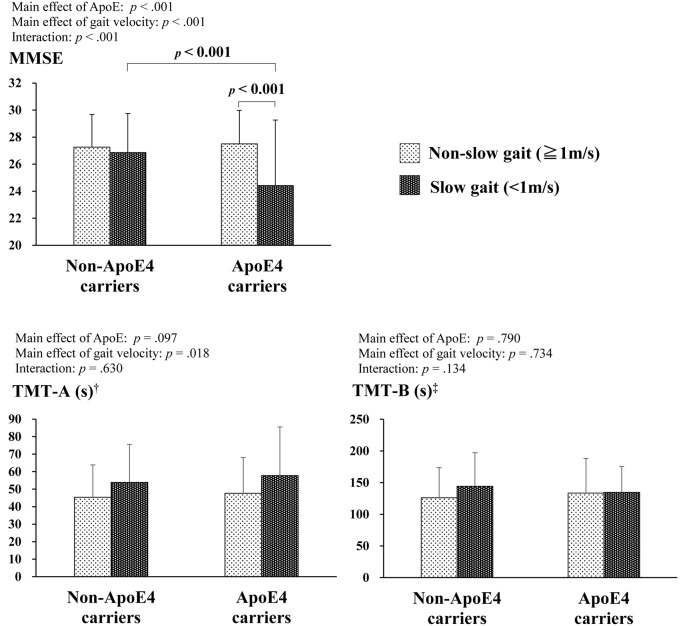
Comparisons of cognitive function by ApoE4 carrier status and gait velocity. ^†^Non-ApoE4 carriers without slow gait: *n* = 792; non-ApoE4 carriers with slow gait: *n* = 104; ApoE4 carriers without slow gait = 148; ApoE4 carriers with slow gait = 18. ^‡^Non-ApoE4 carriers without slow gait: *n* = 719; non-ApoE4 carriers with slow gait: *n* = 84; ApoE4 carriers without slow gait = 138; ApoE4 carriers with slow gait = 14.

Figure 1 shows the results of the two-way ANCOVA with the ApoE and gait velocity factors for the MMSE, TMT-A, and TMT-B. For the TMT, some participants did not complete the assessment because of physical problems (e.g., uncorrected visual defects leading to an inability to visually identify the TMT assessment sheet) and time constraints. The ANCOVA adjusted for covariates showed a significant interaction between the two factors for the MMSE (*F*_(1,1074)_ = 18.4, *p* < 0.001). Subsequent *post hoc* tests demonstrated that ApoE4 carriers with slow gait resulted in lower MMSE scores compared to ApoE4 carriers without slow gait and non-ApoE4 carriers with slow gait. On the other hand, significant effects of gait velocity were observed in the TMT-A only (*F*_(1,1012)_ = 5.6, *p* = 0.018), indicating lower TMT-A scores for slow gait, whereas no significant main effects and interactions between the two factors were observed on the TMT-B.

## Discussion

In a sample of community older adults free of dementia, this study demonstrates for the first time that older ApoE4 carriers who walk slowly present significantly lower MMSE scores. Although caution should be exercised when interpreting a cross-sectional design, our results suggest that the concurrent presence of ApoE4 and slow gait can define a subgroup with the lowest global cognition. This speculation is evident from the results showing that older adults who corresponded with only one of the factors, either ApoE4 or slow gait, did not show significant cognitive impairment.

Emerging evidence shows that ApoE4 is not always associated with cognitive impairment and that other factors may also mediate or modulate the established association between ApoE4 and cognition (Smith et al., 1998; Lupton et al., 2016). Aligned with a previous finding (Jayakody et al., 2019), our results suggest that when the presence of at least one copy of ApoE4 and a slowing gait are overlapped, they may have a synergistic effect on cognitive impairment. Previous and present findings raise the possibility that ApoE4 carriers that exhibit slowing gait may be more likely to develop cognitive decline and progression to dementia. This may point out additional mechanisms that can mediate the association. For instance, white matter disease (WMD) can be expressed by slow gait (Baezner et al., 2008). Future studies should look for the role of WMD in those with ApoE4 and slow gait.

A significant difference in gait velocity between non-ApoE4 and ApoE4 carriers was not observed in the present study. This is consistent with previous studies that show that carriers of ApoE4 among older adults had similar gait velocity to non-carriers (Sakurai and Montero-Odasso, 2017) and that there is no association between ApoE genotype and gait velocity (Vasunilashorn et al., 2013). However, since some studies do show that ApoE4 is associated with a decline in gait velocity (Verghese et al., 2013), there is some disagreement regarding the correlation between ApoE4 and slow gait. A longitudinal study using MCI patients indicated that having the ApoE4 allele was associated with both future decline in gait control (i.e., increased gait variability) and cognitive function, despite lack of a relationship between longitudinal change and gait velocity (Sakurai and Montero-Odasso, 2017). Although this finding suggests that ApoE4 may influence the cognitive processes involved in the control of gait, which eventually influences gait slowing, we did not find significant differences in the parameters of gait variability between non-ApoE4 and ApoE4 carriers. Some factors including brain microvascular damage (i.e., WMD), which results from ApoE4, might mediate the association between ApoE4 and slowing gait (Brickman et al., 2014).

A significant main effect of slow gait was observed on the TMT-A, indicating that older adults who showed slowing gait had reduced abilities in visual search, motor speed skills, and attention, regardless of the presence of ApoE4. This is consistent with previous findings showing no difference in the visuospatial and attention cognitive domains between non-ApoE4 and ApoE4 carriers (Small et al., 2004). Our results suggest that impairment of gait performance may influence these cognitive domains, exacerbating the effects of ApoE4.

Conversely, there were no main effects or interactions of either ApoE4 or slow gait on the TMT-B. This conflicts with previous studies that say ApoE4 and lower gait performance, including slow gait, are associated with deficits in executive functioning (Small et al., 2004; Sakurai et al., 2017). Though lacking significant interaction, non-ApoE4 carriers who did not walk slowly showed decent executive function compared with the other groups (see Figure 1). This is open to further discussion using longitudinal observation.

Strengths of our study include a large sample size diverse in age and sex, as well as being the first study to assess the combined effect of the ApoE4 allele and slowing gait on cognitive function. However, important limitations are mainly grounded in our cross-sectional design, which is known not to infer causation, in the lack of assessments of specific cognitive domains besides executive function that would enrich the associations found, and other potential unmeasured confounders, including subclinical neurological conditions. Furthermore, we excluded dementia patients based on self-reported medical history, which leaves the door open for incorrect selection due to inaccurate self-reports. Further longitudinal studies using medical records may confirm our results. Elucidation of the mechanisms underlying the association between ApoE4 carrier status and low motor performance, as slow gait speed, may point out modifiable factors in populations at risk of dementia.

## Data Availability

The datasets analyzed in this manuscript are not publicly available. Requests to access the datasets should be directed to RS, r_sakurai@hotmail.co.jp.

## Ethics Statement

The studies involving human participants were reviewed and approved by The Tokyo Metropolitan Institute of Gerontology Ethics Board. The patients/participants provided their written informed consent to participate in this study.

## Author Contributions

RS structured the study design, performed statistical analyses, interpreted data, and drafted the manuscript. YW, YO, YT, HiK, HuK, and AK contributed to acquiring all data and interpretation of data. HI, SA, and SS participated in designing this study, acquiring data, and structuring the data set. MM-O provided additions to study design analysis, supervised the interpretation of data, and helped finalize the manuscript.

## Conflict of Interest Statement

The authors declare that the research was conducted in the absence of any commercial or financial relationships that could be construed as a potential conflict of interest.
